# Supplementary material for: High Mitochondrial Protein Expression as a Potential Predictor of Relapse Risk in Acute Myeloid Leukemia Patients with the Monocytic FAB Subtypes M4 and M5
Source: Cancers (Basel). 2023 Dec 19;16(1):8. doi: 10.3390/cancers16010008 (PMC10778527; doi:10.3390/cancers16010008)
Supplement: Supplementary file 1 [file cancers-16-00008-s001.zip › Cancers-AML-differentiation-Supplementary information_revised.pdf]

Table S1. Overview of the 8 FAB-M1/M2 RELAPSE AML patients in this study.

										NPM1	SIGNALING						TUMOR SUPRESSORS				DNA METHYLATION					MYELOID TF		CHROMATIN MODIFICATION				COHESIN		SPLICEOSOME/TR			OTHERS		
Experimental patient code	BM blasts (%)	WBC count (10^9/L)	Gender	Age (years)	Clinical progression	Time to relapse (months after diagnosis)	Survival time (months after diagnosis) (updated on 24/7/18)	FAB classification	Group	NPM1 Ins	FLT3-ITD	FLT3-TKD	NRAS	HRAS	KIT	PTPN11	IKZF1	CDKN2A	PHF6	WT1	DNMT3A	TET2	IDH1	IDH2	KMT2A	RUNX1	CEPBA	GATA2	ASXL1	EZH2	KDM6A	RAD21	STAG2	BCORL1	BCOR	SRSF2	CALR	CSF3R	Cytogenetics
P5	80	43	M	27	CR1 - Relapse - CR2 - SCT	11	274	M2	M1/M2 RELAPSE																														46,XY
P6	95	10	F	66	CR1 - RELAPSE	16	19	M1	M1/M2 RELAPSE																														46,XX
P9	84	27	M	46	CR1 - RELAPSE	27	39	M1	M1/M2 RELAPSE																														46,XY
P12	66	25	F	46	CR1 - Relapse - CR2 - SCT	16	99	M1	M1/M2 RELAPSE																														46,XX,inv(16)(p13q22)
P19	80	16	M	35	CR1 - Relapse - CR2 - SCT	44	135	M2	M1/M2 RELAPSE																														46,XY
P32	95	47	M	62	CR1 - RELAPSE	6	16	M1	M1/M2 RELAPSE																														
P40	95	48	F	63	CR1 - RELAPSE	4	6	M1	M1/M2 RELAPSE																														46,XX,dup(11)(q23.3q23.3)
P48	72	28	M	68	CR1 - RELAPSE	3	23	M1	M1/M2 RELAPSE																														46,XY

The table is adapted from the supplementary Table S1 of our previously published analysis [1]. Gene cell colors indicate: light yellow = wild type; red = mutated; grey = not determined. The grey colored cell on the Cytogenetics column indicate uncertain or not determined data.

TF/TR: transcription factors/repressors; BM: bone marrow; WBC: white blood cells; CR: complete remission; NPM1 Ins: a 4 bp-insertion/duplication; SCT: stem-cell transplantation; ITD: internal tandem duplication; FAB: French-American-British; TKD: tyrosine kinase domain.

Table S2. Overview of the 26 FAB-M4/M5 RELAPSE and RELAPSE-FREE AML patients in this study.

										NPM1	SIGNALING						TUMOR SUPRESSORS				DNA METHYLATION					MYELOID TF		CHROMATIN MODIFICATION				COHESIN		SPLICEOSOME/TR			OTHERS		
Experimental patient code	BM blasts (%)	WBC count $(10^9/L)$	Gender	Age (years)	Clinical progression	Time to relapse (months after diagnosis)	Survival time (months after diagnosis) (updated on 24/7/18)	FAB classification	Group	NPM1 Ins	FLT3-ITD	FLT3-TKD	NRAS	HRAS	KIT	PTPN11	IKZF1	CDKN2A	PHF6	WT1	DNMT3A	TET2	IDH1	IDH2	KMT2A	RUNX1	CEPBA	GATA2	ASXL1	EZH2	KDM6A	RAD21	STAG2	BCORL1	BCOR	SRSF2	CALR	CSF3R	Cytogenetics
P10	89	11	M	57	CR1 - REL_FREE	-	96	M4	M4/M5 REL_FREE																														46,XY
P13	75	22	F	45	CR1 - REL_FREE	-	195	M4	M4/M5 REL_FREE																														46,XX
P14	86	40	F	42	CR1 - REL_FREE	-	150	M5	M4/M5 REL_FREE																														46,XX
P24	50	31	M	53	CR1 - REL_FREE	-	250	M4	M4/M5 REL_FREE																														46,XY
P29	83	71	M	58	CR1 - REL_FREE	-	114	M5	M4/M5 REL_FREE																														46,XY
P31	86	38	F	36	CR1 - REL_FREE	-	232	M5	M4/M5 REL_FREE																														46,XX,t(9;11)(p21;q23)
P36	32	33	M	36	CR1 - REL_FREE	-	70	M4	M4/M5 REL_FREE																														46,XY,inv(16)(p13q22)
P42	90	351	M	43	CR1 - REL_FREE	-	253	M5	M4/M5 REL_FREE																														46,XY,inv(16)
P43	52	50	M	48	CR1 - REL_FREE	-	170	M4	M4/M5 REL_FREE																														46,XY,inv(16)(p13q22)
P44	77	101	M	65	CR1 - REL_FREE	-	109	M5	M4/M5 REL_FREE																														Multiple
P46	83	37	F	60	CR1 - REL_FREE	-	109	M5	M4/M5 REL_FREE																														46,XX
P47	55	9	F	41	CR1 - REL_FREE	-	159	M4	M4/M5 REL_FREE																														46,XX
P49	80	29	M	54	CR1 - REL_FREE	-	174	M5	M4/M5 REL_FREE																														46,XY
P53	50	65	M	48	CR1 - REL_FREE	-	286	M4	M4/M5 REL_FREE																														46,XY
P2	90	19	M	29	CR1 - RELAPSE	12	12	M4	M4/M5 RELAPSE																														46,XY
P11	40	34	F	18	CR1 - Relapse - CR2 - SCT	11	89	M4	M4/M5 RELAPSE																														46,XX,inv(16)(q13q22)
P17	53	14	M	60	CR1 - RELAPSE	6	7	M4	M4/M5 RELAPSE																														46,XY,del(9)(q13q33)[18]
P18	83	58	F	59	CR1 - RELAPSE	8	8	M5	M4/M5 RELAPSE																														46,XX
P22	30	4	M	41	CR1 - RELAPSE	26	42	M4	M4/M5 RELAPSE																														46,XY
P23	97	170	M	46	CR1 - RELAPSE	11	11	M4	M4/M5 RELAPSE																														46,XY
P34	76	14	M	48	CR1 - RELAPSE	6	7	M5	M4/M5 RELAPSE																														46,XY
P37	47	65	F	57	CR1 - RELAPSE	12	12	M4	M4/M5 RELAPSE																														46,XX,inv(16)(13;q22)[5]/46,idem,t(X;6)(p22;p12)[13]/46,XX[2]
P45	95	24	F	61	CR1 - Relapse - CR2 - SCT	12	97	M5	M4/M5 RELAPSE																														46,XX
P50	95	182	F	36	CR1 - RELAPSE	4	12	M4	M4/M5 RELAPSE																														46,XX
P51	95	112	F	32	CR1 - Relapse - CR2 - SCT	12	142	M5	M4/M5 RELAPSE																														46,XX,del(5)(q31q34)[5]/46,XX[15]
P52	25	33	M	62	CR1 - RELAPSE	8	9	M4	M4/M5 RELAPSE																														47,XY,+8[12]/46,XY[13]

The table is adapted from the supplementary Table S1 of our previously published analysis [1]. Gene cell colors indicate: light yellow = wild type; red = mutated; grey = not determined.

TF/TR: transcription factors/repressors; BM: bone marrow; WBC: white blood cells; CR: complete remission; NPM1 Ins: a 4 bp-insertion/duplication; SCT: stem-cell transplantation; ITD: internal tandem duplication; FAB: French-American-British; TKD: tyrosine kinase domain.

Table S3. Differentially expressed proteins by primary AML cells derived from patients without relapse or with a later relapse after intensive therapy: a comparison of REL_M4/5 and REL_F_M4/5 patient proteomes. The information is based on the Gene database (accessed 280823) and selected references from the PubMed database (accessed September 13 2023). The table presents the differentially expressed protein identity (left column), a description of the protein function (middle column) and, keywords with regard to the molecular function and references (right column). These proteins were identified in protein-protein interaction (PPI) analyses of the different REL vs REL_F subgroup comparisons shown in **Figure 5c** of the main text.

	**MITOCHONDRIAL ATP SYNTHESIS PROTON TRANSPORT**	
**Protein id**	**Biological characterization**	**Keywords/References**
ATP5A1 (ATP5F1A) Chromosome 18	*ATP synthase F1 subunit alpha.* The encoded protein is a subunit of mitochondrial ATP synthase that catalyzes ATP synthesis by using an electrochemical gradient of protons across the inner membrane during oxidative phosphorylation. ATP synthase is composed of two linked multi-subunit complexes: the soluble catalytic core, F1, and the membrane-spanning component, Fo, comprising the proton channel. The catalytic portion of mitochondrial ATP synthase consists of 5 different subunits (alpha, beta, gamma, delta, and epsilon) assembled with a stoichiometry of three alpha, three beta and a single chain of the other three. The proton channel consists of three main subunits (a, b, c). This gene encodes the alpha subunit of the catalytic core.	Mitochondria; ATP synthase; Catalytic core subunit/[2-11]
ATP5B (ATP5F1B) Chromosome 12	*ATP synthase F1 subunit beta.* The encoded subunit of mitochondrial ATP synthase that is composed of the soluble catalytic core (F1) and the membrane-spanning (Fo) proton channel. The catalytic portion of mitochondrial ATP synthase consists of 5 different subunits (alpha, beta, gamma, delta, and epsilon) assembled with a stoichiometry of 3 alpha, 3 beta, and a single representative of the other 3. This gene encodes the beta subunit of the catalytic core.	Mitochondria; ATP synthase; Catalytic core subunit/[4-12]
ATP5C1 (ATP5F1C) Chromosome 10	*ATP synthase F1 subunit gamma.* This gene encodes the gamma subunit of the catalytic core of the mitochondrial ATP synthase.	Mitochondria; ATP synthase; Catalytic core subunit/[4-11]
ATP5D (5F1D)	*ATP synthase F1 subunit delta.* This encoded protein is the delta subunit of the catalytic core of the mitochondrial ATP synthase.	Mitochondria; ATP synthase;

Chromosome 19		Catalytic core subunit/[4-11]
ATP5F1 (ATP5PB) Chromosome 1	*ATP synthase peripheral stalk-membrane subunit b.* The encoded protein is the b subunit of the proton channel of the mitochondrial ATP synthase.	Mitochondria; ATP synthase; Proton channel subunit/[4-11]
ATP5H (ATP5PD) Chromosome 17	*ATP synthase peripheral stalk subunit d.* This gene encodes the d subunit of the proton channel complex of mitochondrial ATP synthase.	Mitochondria; ATP synthase; Proton channel subunit/[4-11]
ATP5I Chromosome 4	*ATP synthase, H^+ transporting, mitochondrial Fo complex subunit E.* This encoded protein is the e subunit of the catalytic core of the mitochondrial ATP synthase.	Mitochondria; ATP synthase; Catalytic core subunit/[4-11]
ATP5J (ATP5PE) Chromosome 21	*ATP synthase peripheral stalk subunit F6.* This gene encodes the F6 subunit of the proton channel subunit of mitochondrial ATP synthase. The protein is required for F1 and Fo interactions.	Mitochondria; ATP synthase; Proton channel subunit/[4-11]
ATP5O (ATP5PO) Chromosome 21	*ATP synthase peripheral stalk subunit OSCP.* The encoded protein is a component of the F-type ATPase found in the mitochondrial matrix. F-type ATPases are composed of a catalytic core and a membrane proton channel. The protein appears to be part of the connector linking these two components and may be involved in transmission of conformational changes or proton conductance.	F-type ATPase /[4-11]
MT-ATP8 Mitochondrial	*Mitochondrially encoded ATP synthase 8.* The encoded protein contributes to the mitochondrial proton-transporting ATP synthase activity that is involved in mitochondrial ATP synthesis coupled proton transport. This protein is a part of mitochondrial proton-transporting ATP synthase complex.	Mitochondria; ATP synthase; Proton transport/[4-11]
USMG5 (ATP5MK) Chromosome 10	*ATP synthase membrane subunit k.* The encoded protein is a subunit of the mitochondrial proton-transporting ATP synthase complex.	Mitochondria; ATP synthase; Proton transport/[4-11]

	MITOCHONDRIAL ELECTRON TRANSPORT	
COX15 Chromosome 10	*Cytochrome c oxidase assembly homolog COX15.* Cytochrome c oxidase (COX) is the terminal component of the mitochondrial respiratory chain and catalyzes the electron transfer from reduced cytochrome c to oxygen. This component is a heteromeric complex consisting of 3 catalytic subunits encoded by mitochondrial genes and multiple structural subunits encoded by nuclear genes. The mitochondrially-encoded subunits function in electron transfer, and the nuclear-encoded subunits may function in the regulation and assembly of the complex. This nuclear gene encodes a protein which is not a structural subunit, but may be essential for the biogenesis of COX formation. This protein is predicted to contain 5 transmembrane domains localized in the mitochondrial inner membrane.	Mitochondrial respiratory chain; Cytochrome C oxidase; Electron transfer; Nuclear gene; Transmembrane domains/[13-17]
COX5B Chromosome 2	*Cytochrome c oxidase subunit 5B.* Cytochrome C oxidase (COX) is the terminal enzyme of the mitochondrial respiratory chain. It is a multi-subunit enzyme complex that couples the transfer of electrons from cytochrome c to molecular oxygen and contributes to a proton electrochemical gradient across the inner mitochondrial membrane. The complex consists of 13 mitochondrial- and nuclear-encoded subunits. The mitochondrially-encoded subunits perform the electron transfer and proton pumping activities. The functions of the nuclear-encoded subunits are unknown but they may play a role in the regulation and assembly of the complex. This gene encodes the nuclear-encoded subunit Vb of this enzyme.	Mitochondrial respiratory chain; Cytochrome C oxidase; Electron transfer; Nuclear gene/[13-17]
NDUFB3 Chromosome 2	*NADH:ubiquinone oxidoreductase subunit B3.* This gene encodes an accessory subunit of the mitochondrial membrane respiratory chain NADH dehydrogenase (Complex I) that is the first enzyme in the electron transport chain. This protein localizes to the inner membrane of the mitochondrion.	Mitochondrial respiratory chain Complex I/[18-23]
NDUFC2 Chromosome 11	*NADH:ubiquinone oxidoreductase subunit C2.* The encoded mitochondrial protein is involved in mitochondrial respiratory chain complex I assembly.	Mitochondrial respiratory chain Complex I/[18-23]
NDUFS7 Chromosome 19	*NADH:ubiquinone oxidoreductase core subunit S7.* This gene encodes one of the more than 40 subunits found in complex I of the mitochondrial respiratory chain, the nicotinamide adenine dinucleotide (NADH):ubiquinone oxidoreductase. This complex functions in the transfer of electrons from NADH	Mitochondrial respiratory chain Complex I/[18-23]

	to the respiratory chain, and ubiquinone is believed to be the immediate electron acceptor for the enzyme.	
NDUFV2 Chromosome 18	*NADH:ubiquinone oxidoreductase core subunit V2.* The NADH-ubiquinone oxidoreductase complex (complex I) of the mitochondrial respiratory chain catalyzes the transfer of electrons from NADH to ubiquinone, and consists of at least 43 subunits. The complex is located in the inner mitochondrial membrane. This gene encodes the 24 kDa subunit and is involved in electron transfer.	Mitochondrial respiratory chain Complex I; Electron transfer/[18-23]
UQCRC2 Chromosome 16	*Ubiquinol-cytochrome c reductase core protein 2.* The encoded mitochondrial protein is part of the ubiquinol-cytochrome c reductase complex (also known as complex III) that constitutes a part of the mitochondrial respiratory chain.	Mitochondrial respiratory chain Complex 3/[18,24,25]
UQCRQ Chromosome 5	*Ubiquinol-cytochrome c reductase complex III subunit VII.* The encoded ubiquinone-binding protein is a small core-associated protein that is a subunit of ubiquinol-cytochrome c reductase complex III. This complex is part of the mitochondrial respiratory chain.	Mitochondrial respiratory chain Complex 3/[18,24,25]
	**MITOCHONDRIAL TRANSLATION ELONGATION***	
MRPL10 Chromosome 17	*Mitochondrial ribosomal protein L10.* Mammalian mitochondrial ribosomal proteins are encoded by nuclear genes and help in protein synthesis within the mitochondrion. Mitochondrial ribosomes (mitoribosomes) consist of a small 28S subunit and a large 39S subunit. This gene encodes a 39S subunit protein. Sequence analysis identified three transcript variants that encode two different isoforms.	Mitochondrial ribosome 39S subunit/[26-31]
MRPL11 Chromosome 11	*Mitochondrial ribosomal protein L11.* This nuclear gene encodes a 39S subunit component of the mitochondrial ribosome.	Mitochondrial ribosome 39S subunit/[26-31]
MRPL22 Chromosome 5	*Mitochondrial ribosomal protein L22.* This gene encodes a 39S subunit protein that belongs to the L22 ribosomal protein family.	Mitochondrial ribosome 39S subunit/[26-31]
MRPL54 Chromosome 19	*Mitochondrial ribosomal protein L54.* This gene encodes a 39S subunit protein.	Mitochondrial ribosome 39S subunit/[26-31]

MRPL12 Chromosome 17	*Mitochondrial ribosomal protein L12*. This gene encodes a 39S subunit protein which forms homodimers.	Mitochondrial ribosome 39S subunit/[26-31]
MRPL16 Chromosome 11	*Mitochondrial ribosomal protein L16*. This gene encodes a 39S subunit protein.	Mitochondrial ribosome 39S subunit/[26-31]
MRPL20 Chromosome 1	*Mitochondrial ribosomal protein L20*. This gene encodes a 39S subunit protein.	Mitochondrial ribosome 39S subunit/[26-31]
MRPL24 Chromosome 1	*Mitochondrial ribosomal protein L24*. This gene encodes a 39S subunit protein.	Mitochondrial ribosome 39S subunit/[26-31]
MRPS18A Chromosome 6	*Mitochondrial ribosomal protein S18A*. This gene encodes a 28S subunit protein that belongs to the ribosomal protein S18P family. The primary sequences of the three human mitochondrial S18 proteins are no more closely related to each other than they are to the prokaryotic S18 proteins.	Mitochondrial ribosome 28S subunit/[26-31]
MRPL28 Chromosome 16	*Mitochondrial ribosomal protein L28*. This gene encodes a 39S subunit protein, a part of which was originally isolated by its ability to recognize tyrosinase in an HLA-A24-restricted fashion.	Mitochondrial ribosome 39S subunit/[26-31]
MRPL38 Chromosome 17	*Mitochondrial ribosomal protein L38*. This gene encodes a 39S subunit protein.	Mitochondrial ribosome 39S subunit/[26-31]
MRPL57 Chromosome 13	*Mitochondrial ribosomal protein L57*. This gene encodes a protein which belongs to an undetermined ribosomal subunit and which seems to be specific to animal mitoribosomes.	Mitochondrial ribosomal subunit/[26-31]
MRPS27 Chromosome 5	*Mitochondrial ribosomal protein S27*. This gene encodes a 28S subunit protein that may be a functional partner of the death associated protein 3 (DAP3). Alternative splicing results in multiple transcript variants encoding different isoforms.	Mitochondrial ribosome 28S subunit/[26-31]

	TRANSCRIPTION ELONGATION FROM RNA POLYMERASE II PROMOTER	
SSRP1 Chromosome 11	*Structure specific recognition protein 1.* The encoded protein is a subunit of a heterodimer that, along with SUPT16H, forms chromatin transcriptional elongation factor FACT. FACT interacts with histones H2A/H2B to effect nucleosome disassembly and transcription elongation. FACT and cisplatin-damaged DNA may be crucial to the anticancer mechanism of cisplatin. The protein also functions as a co-activator of the transcriptional activator p63.	Transcriptional elongation; Histone Chemosensitivity/[32-34]
CHD1 Chromosome 5	*Chromodomain helicase DNA binding protein 1.* The CHD protein family is characterized by the presence of chromo (chromatin organization modifier) domains and SNF2-related helicase/ATPase domains. CHD genes alter gene expression possibly by modification of chromatin structure.	Transcription; Chromatin modulation/[32-34]
CTR9 Chromosome 11	*CTR9 homolog, Paf1/RNA polymerase II complex component.* The encoded protein is a component of the PAF1 complex, which associates with RNA polymerase II and functions in transcriptional regulation and elongation. This complex also plays a role in histone modification.	Transcriptional elongation; Histone/[32-34]
RTF1 Chromosome 15	*RTF1 homolog, Paf1/RNA polymerase II complex component.* This locus may represent a gene involved in regulation of transcription elongation and chromatin remodeling, based on studies of similar proteins in other organisms.	Transcription Chromatin/[32-34]
CDC73 Chromosome 1	*Cell division cycle 73.* This gene encodes a tumor suppressor that is involved in transcriptional and post-transcriptional control pathways. The protein is a component of the the PAF complex, which associates with the RNA polymerase II subunit POLR2A and with a histone methyltransferase complex. This protein appears to facilitate the association of 3' mRNA processing factors with actively-transcribed chromatin.	Transcription; Histone methyltransferase/[32-34]
CCNK Chromosome 14	*Cyclin K.* The protein encoded by this gene is a member of the transcription cyclin family. These cyclins may regulate transcription through their association with and activation of cyclin-dependent kinases (CDK) that phosphorylate the C-terminal domain (CTD) of the large subunit of RNA polymerase II. This gene product may play a dual role in regulating CDK and RNA polymerase II activities.	Transcription/[32-34]
CDK13 Chromosome 7	*Cyclin-dependent kinase 13.* The encoded protein is a member of the cyclin-dependent serine/threonine protein kinase family. Members of this family function as master switches in cell cycle control. The exact function of this protein has not yet been determined, but it may play a role in mRNA processing.	Cell cycle regulation; mRNA processing/[32-34]

*There are four mitochondrial translational elongation PPI networks in Figure 5c. Only some of the proteins belonging to these networks are fully described in the table.

References

1. Aasebo, E.; Berven, F.S.; Bartaula-Brevik, S.; Stokowy, T.; Hovland, R.; Vaudel, M.; Doskeland, S.O.; McCormack, E.; Batth, T.S.; Olsen, J.V.; et al. Proteome and Phosphoproteome Changes Associated with Prognosis in Acute Myeloid Leukemia. *Cancers (Basel)* **2020**, *12*, doi:10.3390/cancers12030709.
2. Comelli, M.; Londero, D.; Mavelli, I. Severe energy impairment consequent to inactivation of mitochondrial ATP synthase as an early event in cell death: a mechanism for the selective sensitivity to H2O2 of differentiating erythroleukemia cells. *Free Radic Biol Med* **1998**, *24*, 924-932, doi:10.1016/s0891-5849(97)00373-0.
3. Galber, C.; Acosta, M.J.; Minervini, G.; Giorgio, V. The role of mitochondrial ATP synthase in cancer. *Biol Chem* **2020**, *401*, 1199-1214, doi:10.1515/hsz-2020-0157.
4. Dominguez-Zorita, S.; Cuezva, J.M. The Mitochondrial ATP Synthase/IF1 Axis in Cancer Progression: Targets for Therapeutic Intervention. *Cancers (Basel)* **2023**, *15*, doi:10.3390/cancers15153775.
5. Wang, T.; Ma, F.; Qian, H.L. Defueling the cancer: ATP synthase as an emerging target in cancer therapy. *Mol Ther Oncolytics* **2021**, *23*, 82-95, doi:10.1016/j.omto.2021.08.015.
6. Willers, I.M.; Cuezva, J.M. Post-transcriptional regulation of the mitochondrial H(+)-ATP synthase: a key regulator of the metabolic phenotype in cancer. *Biochim Biophys Acta* **2011**, *1807*, 543-551, doi:10.1016/j.bbabio.2010.10.016.
7. Fiorillo, M.; Ozsvari, B.; Sotgia, F.; Lisanti, M.P. High ATP Production Fuels Cancer Drug Resistance and Metastasis: Implications for Mitochondrial ATP Depletion Therapy. *Front Oncol* **2021**, *11*, 740720, doi:10.3389/fonc.2021.740720.
8. Sennoune, S.R.; Luo, D.; Martinez-Zaguilan, R. Plasmalemmal vacuolar-type H+-ATPase in cancer biology. *Cell Biochem Biophys* **2004**, *40*, 185-206, doi:10.1385/CBB:40:2:185.
9. Chen, H.; Miller, P.W.; Johnson, D.L.; Laribee, R.N. The Ccr4-Not complex regulates TORC1 signaling and mitochondrial metabolism by promoting vacuole V-ATPase activity. *PLoS Genet* **2020**, *16*, e1009046, doi:10.1371/journal.pgen.1009046.
10. Sasazawa, Y.; Futamura, Y.; Tashiro, E.; Imoto, M. Vacuolar H+-ATPase inhibitors overcome Bcl-xL-mediated chemoresistance through restoration of a caspase-independent apoptotic pathway. *Cancer Sci* **2009**, *100*, 1460-1467, doi:10.1111/j.1349-7006.2009.01194.x.
11. von Schwarzenberg, K.; Wiedmann, R.M.; Oak, P.; Schulz, S.; Zischka, H.; Wanner, G.; Efferth, T.; Trauner, D.; Vollmar, A.M. Mode of cell death induction by pharmacological vacuolar H+-ATPase (V-ATPase) inhibition. *J Biol Chem* **2013**, *288*, 1385-1396, doi:10.1074/jbc.M112.412007.
12. Xiao, X.; Yang, J.; Li, R.; Liu, S.; Xu, Y.; Zheng, W.; Yi, Y.; Luo, Y.; Gong, F.; Peng, H.; et al. Deregulation of mitochondrial ATPsyn-beta in acute myeloid leukemia cells and with increased drug resistance. *PLoS One* **2013**, *8*, e83610, doi:10.1371/journal.pone.0083610.

13. Jhas, B.; Sriskanthadevan, S.; Skrtic, M.; Sukhai, M.A.; Voisin, V.; Jitkova, Y.; Gronda, M.; Hurren, R.; Laister, R.C.; Bader, G.D.; et al. Metabolic adaptation to chronic inhibition of mitochondrial protein synthesis in acute myeloid leukemia cells. *PLoS One* **2013**, *8*, e58367, doi:10.1371/journal.pone.0058367.
14. Wiedemann, N.; Pfanner, N. Mitochondrial Machineries for Protein Import and Assembly. *Annu Rev Biochem* **2017**, *86*, 685-714, doi:10.1146/annurev-biochem-060815-014352.
15. Silkjaer, T.; Nyvold, C.G.; Juhl-Christensen, C.; Hokland, P.; Norgaard, J.M. Mitochondrial cytochrome c oxidase subunit II variations predict adverse prognosis in cytogenetically normal acute myeloid leukaemia. *Eur J Haematol* **2013**, *91*, 295-303, doi:10.1111/ejh.12166.
16. Zhang, S.; Zhao, Y.; Heaster, T.M.; Fischer, M.A.; Stengel, K.R.; Zhou, X.; Ramsey, H.; Zhou, M.M.; Savona, M.R.; Skala, M.C.; et al. BET inhibitors reduce cell size and induce reversible cell cycle arrest in AML. *J Cell Biochem* **2019**, *120*, 7309-7322, doi:10.1002/jcb.28005.
17. Passaniti, A.; Kim, M.S.; Polster, B.M.; Shapiro, P. Targeting mitochondrial metabolism for metastatic cancer therapy. *Mol Carcinog* **2022**, *61*, 827-838, doi:10.1002/mc.23436.
18. Tang, J.X.; Thompson, K.; Taylor, R.W.; Olahova, M. Mitochondrial OXPHOS Biogenesis: Co-Regulation of Protein Synthesis, Import, and Assembly Pathways. *Int J Mol Sci* **2020**, *21*, doi:10.3390/ijms21113820.
19. Damm, F.; Bunke, T.; Thol, F.; Markus, B.; Wagner, K.; Gohring, G.; Schlegelberger, B.; Heil, G.; Reuter, C.W.; Pullmann, K.; et al. Prognostic implications and molecular associations of NADH dehydrogenase subunit 4 (ND4) mutations in acute myeloid leukemia. *Leukemia* **2012**, *26*, 289-295, doi:10.1038/leu.2011.200.
20. Morgan, M.A.; Markus, B.; Hermkens, M.; Damm, F.; Reinhardt, D.; Zimmermann, M.; Thol, F.; Bunke, T.; Bogoeva, D.; Reuter, C.W.; et al. NADH dehydrogenase subunit 4 variant sequences in childhood acute myeloid leukaemia. *Br J Haematol* **2013**, *161*, 891-895, doi:10.1111/bjh.12298.
21. Baccelli, I.; Gareau, Y.; Lehnertz, B.; Gingras, S.; Spinella, J.F.; Corneau, S.; Mayotte, N.; Girard, S.; Frechette, M.; Blouin-Chagnon, V.; et al. Mubritinib Targets the Electron Transport Chain Complex I and Reveals the Landscape of OXPHOS Dependency in Acute Myeloid Leukemia. *Cancer Cell* **2019**, *36*, 84-99 e88, doi:10.1016/j.ccell.2019.06.003.
22. Kuang, Y.; Peng, C.; Dong, Y.; Wang, J.; Kong, F.; Yang, X.; Wang, Y.; Gao, H. NADH dehydrogenase subunit 1/4/5 promotes survival of acute myeloid leukemia by mediating specific oxidative phosphorylation. *Mol Med Rep* **2022**, *25*, doi:10.3892/mmr.2022.12711.
23. Sousa, J.S.; D'Imprima, E.; Vonck, J. Mitochondrial Respiratory Chain Complexes. *Subcell Biochem* **2018**, *87*, 167-227, doi:10.1007/978-981-10-7757-9_7.
24. Li, G.; Qin, Y. Mitochondrial translation factor EF4 regulates oxidative phosphorylation complexes and the production of ROS. *Free Radic Res* **2018**, *52*, 1250-1255, doi:10.1080/10715762.2018.1479063.
25. Zhang, Y.; Luo, T.; Ding, X.; Chang, Y.; Liu, C.; Zhang, Y.; Hao, S.; Yin, Q.; Jiang, B. Inhibition of mitochondrial complex III induces differentiation in acute myeloid leukemia. *Biochem Biophys Res Commun* **2021**, *547*, 162-168, doi:10.1016/j.bbrc.2021.02.027.
26. Lightowlers, R.N.; Rozanska, A.; Chrzanowska-Lightowlers, Z.M. Mitochondrial protein synthesis: figuring the fundamentals, complexities and complications, of mammalian mitochondrial translation. *FEBS Lett* **2014**, *588*, 2496-2503, doi:10.1016/j.febslet.2014.05.054.
27. Mai, N.; Chrzanowska-Lightowlers, Z.M.; Lightowlers, R.N. The process of mammalian mitochondrial protein synthesis. *Cell Tissue Res* **2017**, *367*, 5-20, doi:10.1007/s00441-016-2456-0.

28. Ott, M.; Amunts, A.; Brown, A. Organization and Regulation of Mitochondrial Protein Synthesis. *Annu Rev Biochem* **2016**, *85*, 77-101, doi:10.1146/annurev-biochem-060815-014334.
29. Rudler, D.L.; Hughes, L.A.; Viola, H.M.; Hool, L.C.; Rackham, O.; Filipovska, A. Fidelity and coordination of mitochondrial protein synthesis in health and disease. *J Physiol* **2021**, *599*, 3449-3462, doi:10.1113/JP280359.
30. Wang, F.; Zhang, D.; Zhang, D.; Li, P.; Gao, Y. Mitochondrial Protein Translation: Emerging Roles and Clinical Significance in Disease. *Front Cell Dev Biol* **2021**, *9*, 675465, doi:10.3389/fcell.2021.675465.
31. Skrtic, M.; Sriskanthadevan, S.; Jhas, B.; Gebbia, M.; Wang, X.; Wang, Z.; Hurren, R.; Jitkova, Y.; Gronda, M.; Maclean, N.; et al. Inhibition of mitochondrial translation as a therapeutic strategy for human acute myeloid leukemia. *Cancer Cell* **2011**, *20*, 674-688, doi:10.1016/j.ccr.2011.10.015.
32. Habbane, M.; Montoya, J.; Rhouda, T.; Sbaoui, Y.; Radallah, D.; Emperador, S. Human Mitochondrial DNA: Particularities and Diseases. *Biomedicines* **2021**, *9*, doi:10.3390/biomedicines9101364.
33. Saravanan, S.; Lewis, C.J.; Dixit, B.; O'Connor, M.S.; Stolzing, A.; Boominathan, A. The Mitochondrial Genome in Aging and Disease and the Future of Mitochondrial Therapeutics. *Biomedicines* **2022**, *10*, doi:10.3390/biomedicines10020490.
34. Bralha, F.N.; Liyanage, S.U.; Hurren, R.; Wang, X.; Son, M.H.; Fung, T.A.; Chingcuanco, F.B.; Tung, A.Y.; Andreazza, A.C.; Psarianos, P.; et al. Targeting mitochondrial RNA polymerase in acute myeloid leukemia. *Oncotarget* **2015**, *6*, 37216-37228, doi:10.18632/oncotarget.6129.

MITOCHONDRIAL RIBOSOMAL PROTEINS

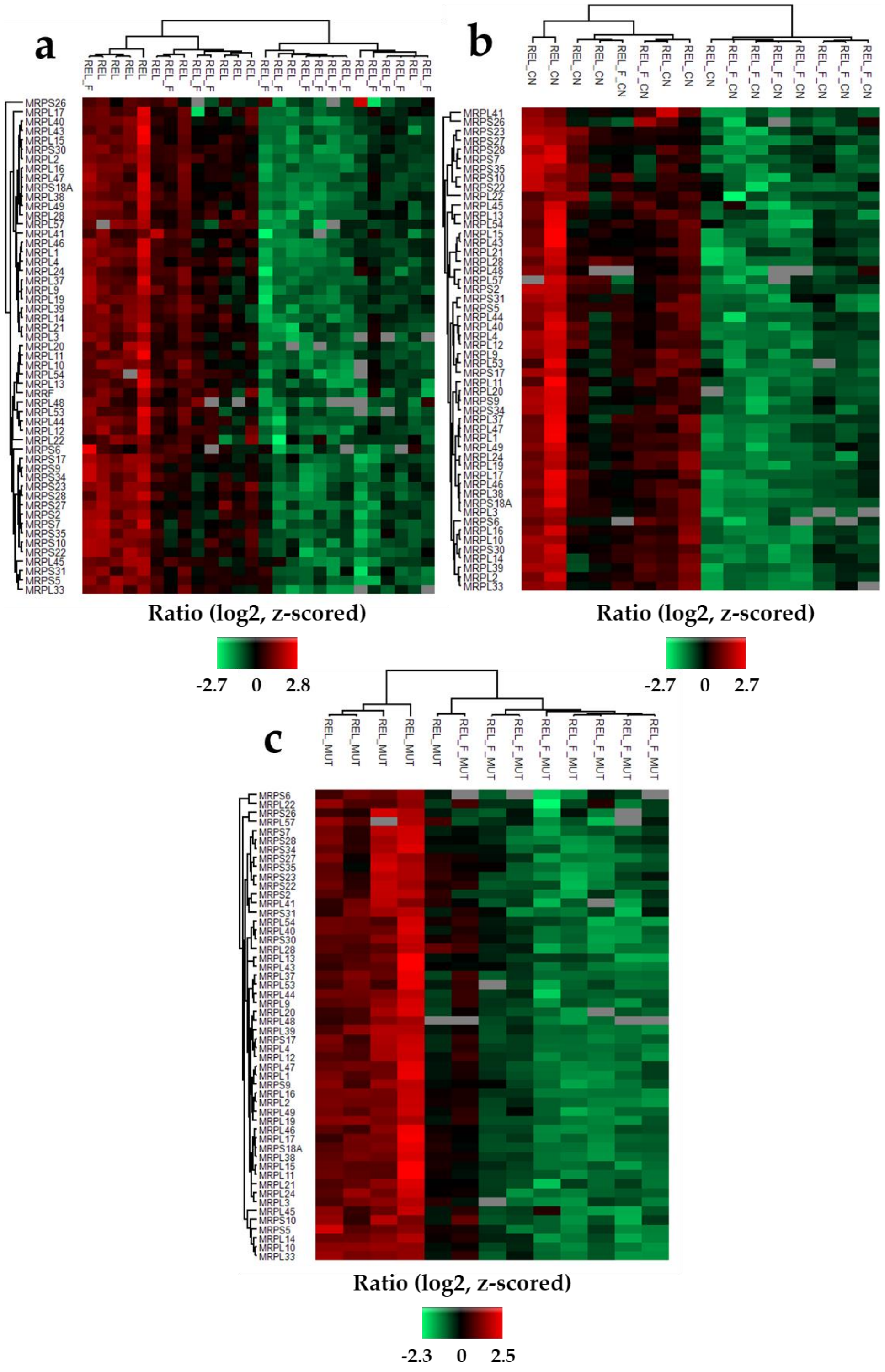

Figure S1. Hierarchical clustering of all M4/M5-FAB classified relapse (REL) and relapse-free (REL_F) patients (**a**), M4/M5-FAB classified REL and REL_F patients with normal cytogenetics -CN- (**b**), and M4/M5-FAB classified REL and REL_F patients with *NPM1* Ins mutation -MUT- **(c)**, based on the expression of mitochondrial ribosomal proteins with a significantly different regulation identified by ANOVA.

MITOCHONDRIAL ATP SYNTHASE

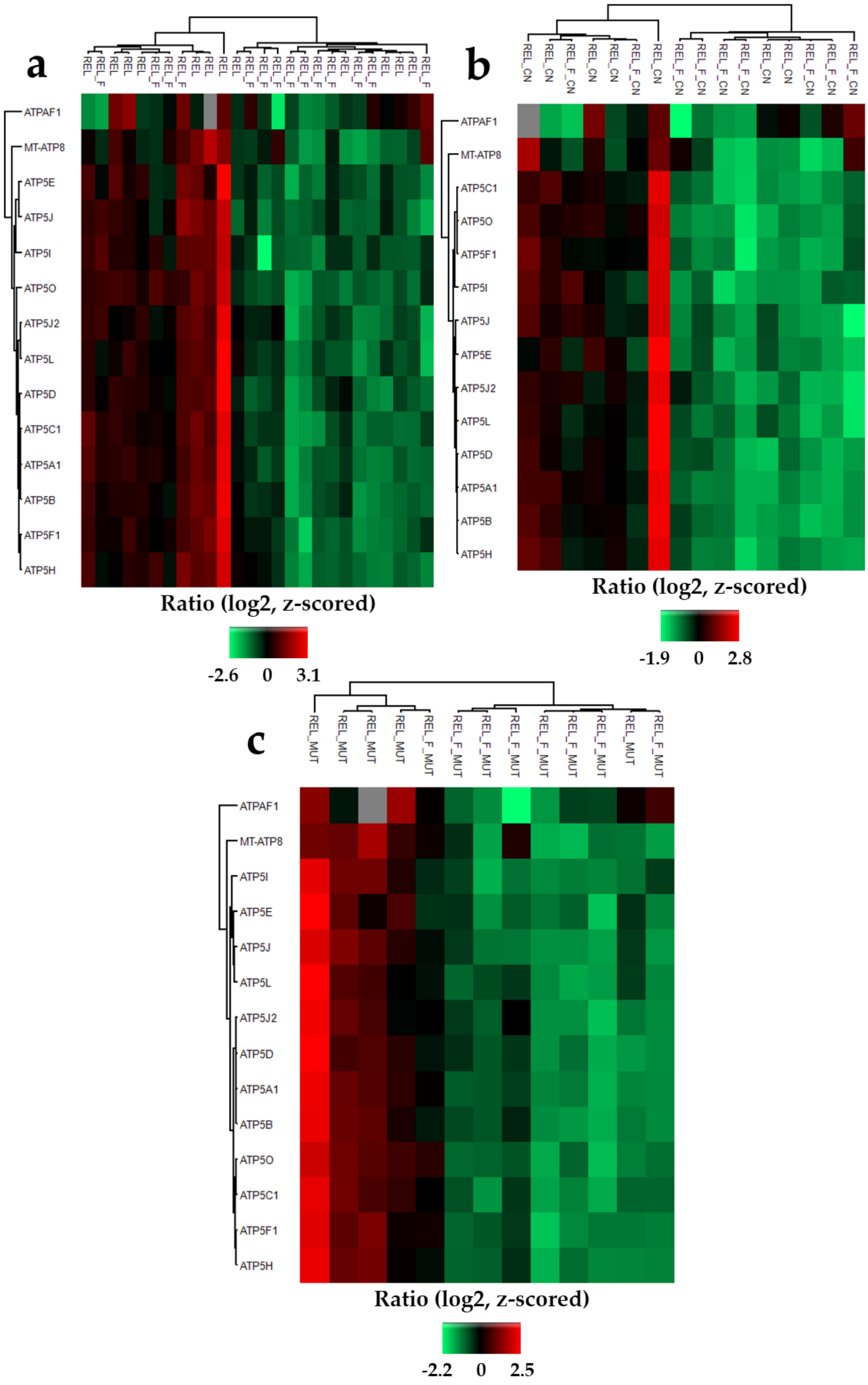

Figure S2. Hierarchical clustering of all M4/M5-FAB classified relapse (REL) and relapse-free (REL_F) patients (**a**), M4/M5-FAB classified REL and REL_F patients with normal cytogenetics -CN- (**b**), and M4/M5-FAB classified REL and REL_F patients with *NPM1* Ins mutation -MUT- (**c**), based on the expression of mitochondrial ATP synthase proteins with a significantly different regulation identified by ANOVA.

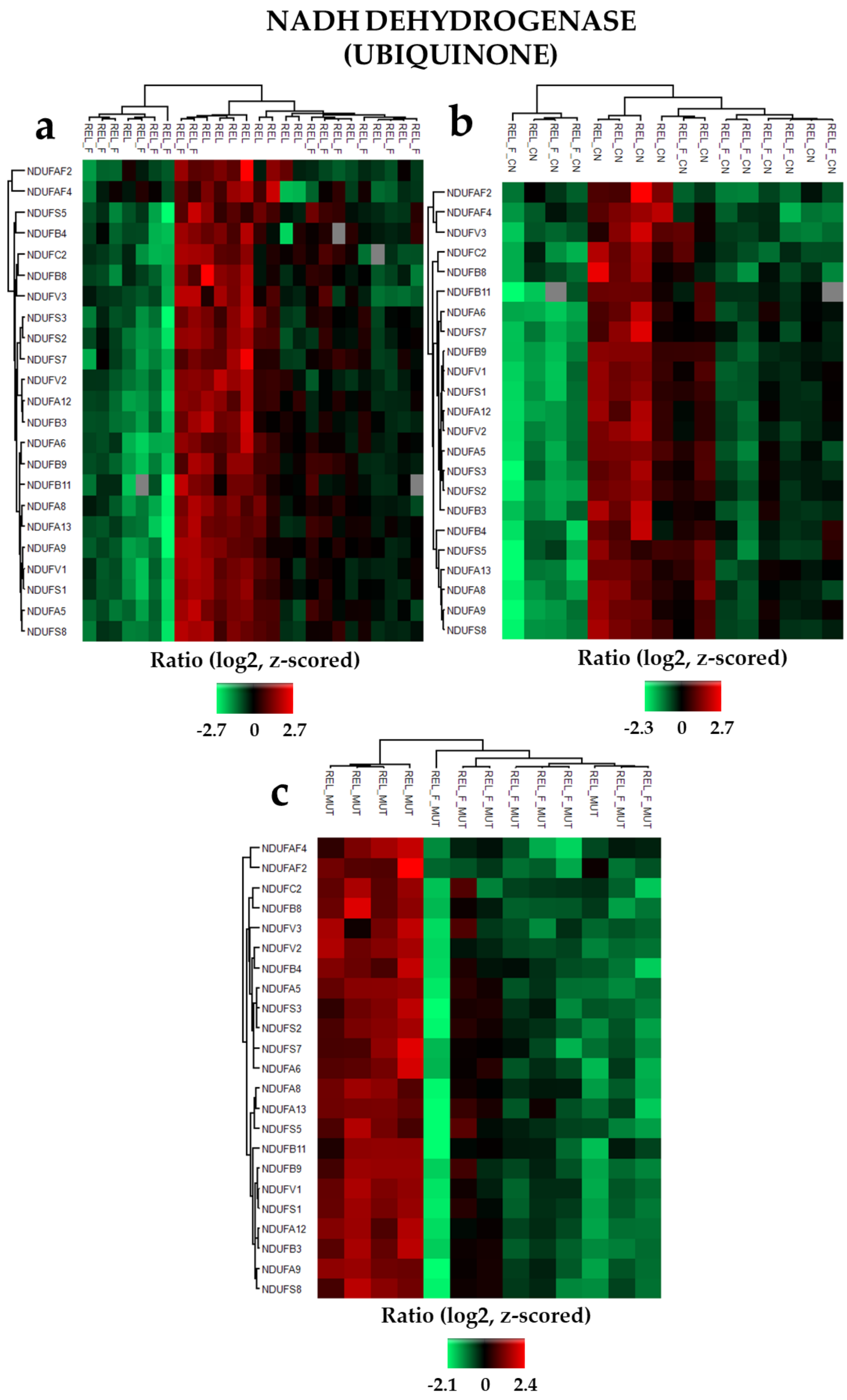

Figure S3. Hierarchical clustering of all M4/M5-FAB classified relapse (REL) and relapse-free (REL_F) patients (**a**), M4/M5-FAB classified REL and REL_F patients with normal cytogenetics -CN- (**b**), and M4/M5-FAB classified REL and REL_F patients with *NPM1* Ins mutation -MUT- (**c**), based on the expression of NADH dehydrogenase (ubiquinone) proteins with a significantly different regulation identified by ANOVA.
